# Impact of ventricular tachycardia ablation in the setting of electrical storm based on patient risk profile

**DOI:** 10.1093/europace/euaf188

**Published:** 2025-08-20

**Authors:** Sandro Ninni, Charles Guenancia, Ophélie Bourdrel, Rayan Mohammed, Donovan Decaudin, Cédric Klein, Alexandre Salaun, Ruxandra Sava, Amine Tazibet, Soundous M'Rabet, Pierre Grégoire Guinot, Pierre Groussin, Antoine Da Costa, Dominique Pavin, Didier Klug, Raphaël Martins, Karim Benali

**Affiliations:** Department of Cardiology, Lille University Hospital, 59000 Lille, France; Department of Cardiology, Dijon University Hospital, Dijon, France; Department of Cardiology, Lille University Hospital, 59000 Lille, France; Section of Cardiac Electrophysiology, Saint-Etienne University Hospital, Saint-Etienne, France; Section of Cardiac Electrophysiology, Rennes University Hospital, Rennes, France; Department of Cardiology, Lille University Hospital, 59000 Lille, France; Department of Cardiology, Dijon University Hospital, Dijon, France; Section of Cardiac Electrophysiology, Saint-Etienne University Hospital, Saint-Etienne, France; Department of Cardiology, Lille University Hospital, 59000 Lille, France; Department of Cardiology, Dijon University Hospital, Dijon, France; Department of Cardiology, Dijon University Hospital, Dijon, France; Section of Cardiac Electrophysiology, Rennes University Hospital, Rennes, France; Section of Cardiac Electrophysiology, Saint-Etienne University Hospital, Saint-Etienne, France; Section of Cardiac Electrophysiology, Rennes University Hospital, Rennes, France; Department of Cardiology, Lille University Hospital, 59000 Lille, France; Section of Cardiac Electrophysiology, Rennes University Hospital, Rennes, France; INSERM-LTSI, Rennes U1099, France; Section of Cardiac Electrophysiology, Saint-Etienne University Hospital, Saint-Etienne, France; IHU Liryc, Electrophysiology and Heart Modeling Institute, Bordeaux, France; Haut-Leveque University Hospital, Bordeaux, France

**Keywords:** Ventricular tachycardia, Catheter ablation, Heart failure, Electrical storm

## Abstract

**Aims:**

Catheter ablation (CA) plays a central role in the management of electrical storm (ES). PAINESD and iVT are two validated scores commonly used to assess periprocedural risk in patients undergoing ventricular tachycardia (VT) ablation. This study aimed to evaluate the association between CA and mortality in ES patients stratified by PAINESD and iVT risk scores.

**Methods and results:**

We included 606 patients admitted for ES across four French centres. Risk was assessed using PAINESD and iVT scores. Mortality at 1 year was compared according to risk group and CA status. Baseline differences were adjusted using inverse probability of treatment weighting (IPTW) based on predefined clinical variables. Forty-one per cent of patients were classified as high-risk using the PAINESD score, and 39.4% using the iVT score. Catheter ablation was performed in 42.4% of the cohort, including 39.4% of hi-PAINESD and 35.5% of hi-iVT patients. After adjustment, CA was associated with lower 1-year mortality in both high- and low-risk groups (adjusted HR: 0.42 [95% CI: 0.26–0.66], *P* = 0.0002 for hi-PAINESD; 0.31 [0.18–0.53], *P* < 0.0001 for hi-iVT). Inverse probability of treatment weighting-weighted models yielded consistent results. In exploratory interaction analyses, the iVT score—but not PAINESD—identified high-risk patients who may derive earlier survival benefit from VT ablation.

**Conclusion:**

In patients with electrical storm, VT ablation was associated with lower 1-year mortality across both low- and high-risk profiles. The iVT score may help identify high-risk patients who derive earlier benefit from ablation.

## Graphical Abstract

**Graphical Abstract:**
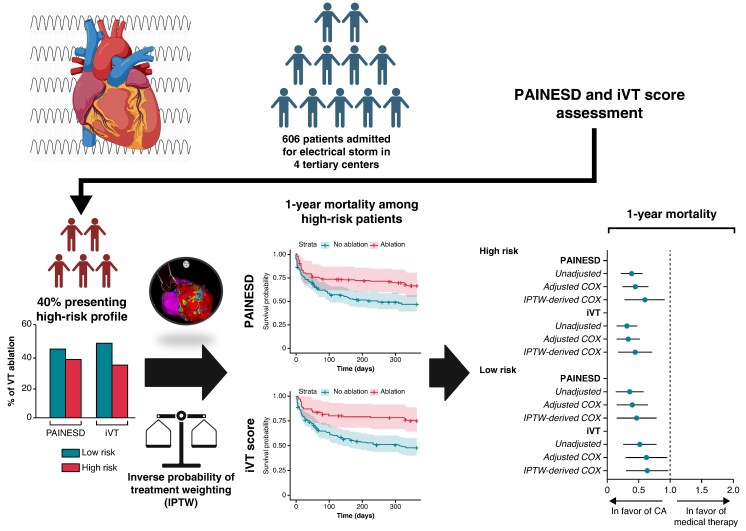

What’s new?

- Catheter ablation is a cornerstone in the management of patients presenting electrical storm (ES). PAINESD and iVT are two scoring tools commonly used to assess periprocedural risks associated with ventricular tachycardia (VT) ablation. The effectiveness of VT ablation in ES patients with a high risk of acute haemodynamic decompensation (AHD) and mortality remains uncertain.
- Approximately 40% of ES patients are classified as high-risk for AHD or postoperative mortality, whether assessed by PAINESD or iVT scores. VT ablation is associated with a significantly lower occurrence of 1-year mortality across both low- and high-risk groups. Notably, the iVT score may help identify high-risk patients who derive an earlier survival benefit from ablation.

## Introduction

Electrical storm (ES) is a life-threatening condition and a major cardiological emergency, defined by the occurrence of at least three sustained ventricular arrhythmias within 24 h.^1^ Despite advancements in the management of structural heart diseases, the incidence of ES remains significant, ranging from 10% to 30% in patients with implantable cardioverter-defibrillators (ICDs) for secondary prevention. ES is strongly associated with increased mortality, reaching up to 40% at one year.^2^ Patients presenting with ES generally require intensive care unit admission and multidisciplinary management through multimodal therapeutic approaches.

Catheter ablation (CA) has become a cornerstone for treating recurrent ventricular tachycardia (VT) despite optimal medical treatment.^3^ Although significant progress was made in VT ablation techniques over the last decade, postoperative mortality remains significant, particularly for patients at high risk of acute haemodynamic decompensation (AHD).^4^ When performed in the context of ES, VT ablation is associated with a higher postoperative mortality and an increased rate of VT recurrences.^4^ The PAINESD and iVT scores are two scoring tools that allow clinicians to estimate postoperative mortality in patients undergoing VT ablation, stratifying them into low vs. high risk for postoperative mortality.^5,6^ Our group recently provided additional data suggesting a benefit of VT ablation in the setting of ES based on a propensity matching approach in a large cohort.^7^ However, the effect of VT ablation according to patient risk stratification has not been reported, particularly in those with the highest risk profiles. Since high postoperative mortality was reported among high-risk patients, the relevance of VT ablation vs. medical treatment can be questioned.

Thus, the aim of this study was to investigate the prognostic impact of VT ablation in patients exhibiting high-risk profiles as assessed by the PAINESD and the iVT score.

## Methods

### Study population

This multicentre retrospective study included all consecutive patients admitted in the intensive care units of four French tertiary centres (Saint-Etienne, Rennes, Lille and Dijon university hospital centres) for the management of ES in the context of structural heart disease between the 1 January 2010 and the 1 March 2023. ES was consensually defined as the occurrence of at least 3 or more distinct episodes of sustained ventricular arrhythmias [ventricular tachycardia (VT) or ventricular fibrillation (VF)] within 24 h or incessant ventricular arrhythmias for more than 12 h.^8,9^ In patients with ICD, ES was defined by the occurrence of ≥3 appropriate device therapies within 24 h, separated by at least 5 min.^8–10^ For patients with several admissions for ES over the observation period, only the first admission was considered for the primary analysis. Adults under legal protection or aged <18 years old were not included. Patients with ES occurring without underlying structural heart disease (e.g. channelopathy, idiopathic VF, and drug-induced torsade de pointes) were also excluded from the analyses. This study was approved by the local ethic committee and patients gave their informed consent to participate in the study.

### Data collection

Baseline data, including demographic characteristics, past medical history, type of cardiomyopathy, left ventricular ejection fraction (LVEF), history of ventricular arrhythmias prior to the ES, history of previous CA, presence of an ICD and treatment at the time of ES admission were collected from medical files for all enrolled patients. In-hospital data were collected including clinical presentation, laboratory parameters, ES tolerance, and ES treatment (type of antiarrhythmic drugs used, use of deep sedation and intubation, use of invasive haemodynamic support, use of sympathetic blockade). Cardiogenic shock was defined as a systolic blood pressure < 90 mmHg with appropriate fluid resuscitation with clinical and laboratory evidence of end-organ damage and the requirement for inotropic drug.^11^ Occurrence of in-hospital death, as well as date and causes of death after discharge, were also collected using data from each tertiary centre. Medical records from facilities outside of the main centres were also reviewed through a linked electronic medical record system or by contacting the referring physicians.

### PAINESD and iVT score assessment

The PAINESD score includes the following components: history of respiratory diseases (5 points); age > 60 years (3 points); history of ischaemic cardiomyopathy (6 points); NYHA > II (6 points); LVEF < 25% (3 points); ES (5 points); and diabetes (3 points). Patients were considered as high risk for PAINESD ≥ 15. The iVT score was computed using the online calculator https://www.vtscore.org.

### Follow-up

Outpatient follow-up after discharge was performed according to local hospital protocol. Telephone interviews were also performed in some patients to confirm survival when information was not available from other sources.

### Study endpoints

The primary clinical outcome was 1-year mortality following ES.

### Statistical analysis

Continuous variables were tested for normality with the Shapiro test. Continuous variables with Gaussian distribution are given as mean ± SD. Continuous variables with non-Gaussian distribution are given as median (IQR). Categorical variables are given as percentages of individuals. Bivariate comparisons were performed using the *t*-test for normally distributed continuous variables or the Mann–Whitney *U* test for not normally distributed variables. Bivariate comparisons of categorical variables were done with the χ^2^ test.

To assess the impact of VT ablation on primary outcome following ES, a log-rank test was performed as a first step and unadjusted hazard ratios (HRs) (95% confidence intervals [CIs]) were computed. Univariate Cox analysis was performed to identify variables of interest for multivariable adjustment. A multivariable Cox model was then performed and included competing variables associated with the primary outcome in univariate analysis and adjusted HRs (95% [CIs]) were computed. A value of *P* < 0.05 was judged to be statistically significant. To further investigate the effect of VT ablation across different risk profiles, inverse probability of treatment weighting (IPTW) was applied, based on propensity scores derived from a logistic regression model including predefined clinically relevant variables: age, NYHA class, acute pulmonary oedema or cardiogenic shock, history of electrical storm, LVEF, catecholamine use, prior ICD implantation, and atrial fibrillation. Baseline characteristics between groups were assessed using standardized mean differences (SMD), with a threshold of 0.1 indicating a meaningful imbalance. Stabilized IPTW weights were trimmed at the 1st and 99th percentiles to mitigate the influence of extreme values. These weights were applied to compute weighted means and standard deviations for continuous variables, and weighted proportions for categorical variables, using the survey package in R. Survival analyses were conducted using Cox proportional hazards models. Next, we created discrete follow-up endpoints censoring each patient at 90, 180, and 365 days post-admission. For each horizon we fit an IPTW-weighted Cox proportional hazards model of time to death that included main effects for ablation and risk group and their multiplicative interaction. The interaction *P*-value at each time point tested whether the HR for ablation differed significantly between low- and high-risk patients. Kaplan–Meier survival curves were generated for both unadjusted and IPTW-weighted cohorts. All statistical analyses were performed with R (version 4.5.1).

## Results

### Population baseline characteristics

**Figure 1:**
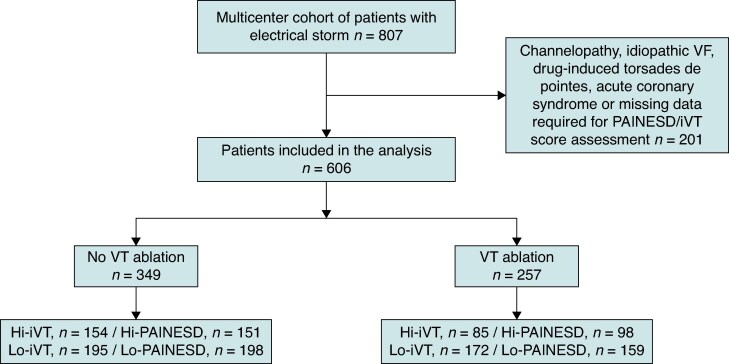
Population flow chart.

A total of 807 patients admitted for ES between 2010 and 2023 in the four participating centres were screened for inclusion. Among them, 201 patients presented acute coronary syndrome, channelopathy, idiopathic VF, drug-induced torsades de pointes, or had missing data required for PAINESD/iVT score assessment. A total of 606 patients met the inclusion criteria and were considered for analysis in the study. *Figure 1* provides the study flow chart. Median age was 67 [59; 74], 14.6% were female, 64.9% had ischaemic cardiomyopathy, and median LVEF was 30% [20; 42]. Regarding management of ES, 83.6% of patients received amiodarone, 22.8% of patients underwent deep sedation, and 1.7% underwent stellate ganglion block. In addition, 23.4% of patients required catecholamine infusion due to haemodynamic instability. The baseline characteristics of the population are reported in *Table 1*.

**Table 1:** Baseline characteristics according to ventricular tachycardia ablation in the entire cohort

	Overall population *n* = 606	No ablation *n* = 349	Ablation *n* = 257	*P*-value
Gender (male)	520 (85.4%)	289 (82.8%)	231 (89.9%)	**0**.**0188**
Age	67 [59; 74]	67 [56; 76]	67 [59; 72]	**0**.**0344**
BMI (kg/m^2^)	27.08 [24.22; 30.45]	26.44 [23.56; 30.10]	27.57 [24.76; 30.86]	**0**.**0205**
Associated comorbidities				
Hypertension	337 (55.6%)	188 (53.9%)	149 (58%)	0.3559
Diabetes	170 (28.1%)	107 (30.7%)	63 (24.5%)	0.1158
Dyslipidaemia	349 (57.6%)	201 (57.6%)	148 (57.6%)	0.9348
Active smokers	247 (40.8%)	140 (40.1%)	107 (41.6%)	0.7698
Peripheral artery disease	85 (14%)	50 (14.3%)	35 (13.6%)	0.8968
History of stoke	64 (10.6%)	39 (11.2%)	25 (9.7%)	0.6606
Chronic kidney disease	130 (21.5%)	82 (23.5%)	48 (19.7%)	0.1842
History of atrial fibrillation	265 (43.7%)	147 (42.1%)	118 (45.9%)	0.3966
Chronic respiratory disease	71 (11.7%)	40 (11.5%)	31 (12.1%)	0.9207
History of ES	109 (18%)	50 (14.3%)	59 (23%)	**0**.**0086**
History of VT ablation	89 (14.7%)	40 (11.4%)	49 (19.1%)	**0**.**0125**
Underlying cardiomyopathy				
Ischaemic cardiomyopathy	393 (64.9%)	221 (63.3%)	172 (66.9%)	0.4055
Non-ischaemic cardiomyopathy	213 (35.1%)	128 (36.6%)	85 (33%)	
LVEF (%)	30 [20; 42]	30 [20; 40]	35 [25; 45]	**0**.**0193**
NYHA III–IV	163 (29.5%)	107 (31.2%)	56 (23.5%)	**0**.**0105**
Baseline treatments				
Amiodarone	185 (30.6%)	93 (26.6%)	92 (35.9%)	**0**.**0182**
Betablockers	494 (83%)	266 (77.6%)	228 (90.5%)	**0**.**0001**
ACE inhibitor	289 (48.6%)	154 (44.9%)	135 (53.6%)	**0**.**0446**
ARB	60 (10.1%)	35 (10.2%)	25 (9.9%)	0.9806
Aldosterone antagonist	195 (32.3%)	99 (28.4%)	96 (37.6%)	**0**.**0203**
ICD	412 (68%)	209 (59.9%)	203 (79%)	**<0.0001**
CRT	135 (22.3%)	83 (23.8%)	52 (20.2%)	0.3478
Admission parameters				
Acute pulmonary oedema or cardiogenic shock	99 (16.3%)	80 (22.9%)	19 (7.4%)	**<0.0001**
Creatinine (μg/dL)	114 [88; 150]	114 [88; 155]	106 [88; 141]	0.0999
Management of ES				
Amiodarone	506 (83.6%)	294 (84.5%)	212 (82.8%)	0.6610
Betablockers	228 (37.6%)	108 (30.9%)	120 (46.6%)	0.1328
Cathecholamines	142 (23.4%)	102 (29.2%)	40 (15.6%)	**0**.**0001**
Dialysis	26 (4.3%)	20 (5.7%)	6 (2.3%)	0.0664
Deep sedation	138 (22.8%)	81 (23.2%)	57 (22.2%)	0.8408
Stellate ganglion block	10 (1.7%)	5 (1.4%)	5 (2%)	0.8562

Continuous quantitative variables with a non-normal distribution are presented as median [interquartile range]. Categorical variables are expressed as the number of patients (percentage). Bold values indicate statistically significant *P*-values (*P* < 0.05).

ACE inhibitor, angiotensin-converting enzyme inhibitor; ARB, angiotensin II receptor blocker; BMI, body mass index; CRT, cardiac resynchronization therapy; ES, electrical storm; VT, ventricular tachycardia; ICD, implantable cardioverter-defibrillator; LVEF, left ventricular ejection fraction.

A total of 257 patients (42.4%) underwent VT ablation. Patients who underwent VT ablation were younger, had higher BMI, higher rate of history of ES or VT ablation, higher LVEF, and lower NHYA status (*Table 1*).

### Risk stratification according to PAINESD and iVT scores

**Figure 2:**
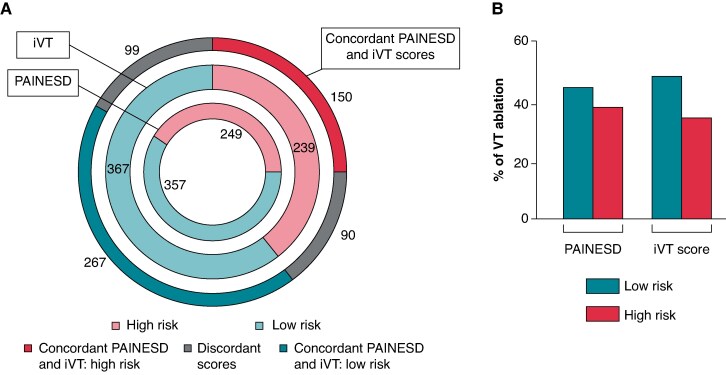
Risk stratification according to iVT and PANESD scores and risk profiles overlap. (*A*) Concordance of PAINESD and iVT scores. Inner circle: risk stratification according to the PAINESD score. Middle circle: risk stratification according to the iVT score. Outer circle: concordance between the two scores, patients classified as high risk by both scores, patients classified as low risk by both scores, discordance between the scores. (*B*) Proportion of patients undergoing VT ablation according to risk stratification based on the PAINESD and the iVT scores.

Forty-one per cent of patients were classified as high risk for VT ablation based on the PAINESD score (hi-PAINESD), and 39.4% were considered high risk according to the iVT score (hi-iVT), with 24.7% of patients identified as high risk by both scores (*Figure 2A*). As expected, high-risk patients tended to be older, more frequently had ischaemic cardiomyopathy, exhibited lower left ventricular ejection fraction (LVEF), presented more severe NYHA status, had more critical conditions upon admission, and required catecholamines more often. Baseline characteristics according to risk profile are reported as supplementary materials (Supplementary material online, *Tables S1* and *S2*).

The proportion of high-risk patients undergoing VT ablation was 39.4% for hi-PAINESD and 35.5% for hi-iVT. Among patients stratified as low-intermediate risk based on the PAINESD score (lo-PAINESD) 43% underwent VT ablation and 47% in patients stratified as low risk based on iVT score (lo-iVT) (*Figure 2B*). Among patients who underwent VT ablation, the mean procedure time was 186 ± 55 min. In 42 cases, epicardial approach was performed. The rate of postoperative complications was 5.4%, including 2 tamponade, 7 ischaemic strokes, and 3 arteriovenous fistula. No procedure-related death occurred (see Supplementary material online, *Table S3*).

### VT ablation according to risk profile

High-risk patients who underwent ablation were more likely to have a history of ES, while they were less likely to require catecholamine support. Hi-PAINESD patients who underwent VT ablation also had higher LVEF and were more likely to have an ICD prior to admission (*Tables 2* and *3*).

**Table 2:** Baseline characteristics according to VT ablation in high-risk patients based on the PAINESD score

Variable	Overall population (*n* = 249)	No ablation (*n* = 151)	Ablation (*n* = 98)	*P*-value	SMD (pre-IPTW)	No ablation IPTW	Ablation IPTW	SMD (post-IPTW)
Male gender	223 (89.6%)	134 (88.7%)	89 (90.8%)	0.7559	−0.006	90%	91%	−0.02
Age, years	70 [68; 71]	71 [65; 77]	68 [65; 87]	0.236	−0.09	70 ± 8.9	70 ± 7.5	0.033
BMI (kg/m²)	27.4 [26.7; 28]	26.8 [24.2; 30.8]	27.7 [25.3; 31.8]	0.1211	0.12	28.3 ± 5.3	27.6 ± 5.1	−0.03
Associated comorbidities								
Hypertension	171 (68.7%)	100 (66.2%)	71 (72.4%)	0.3710	0.06	65%	72%	0.09
Diabetes	130 (52.2%)	83 (55%)	47 (48%)	0.3413	−0.09	62%	48%	−0.08
Dyslipidaemia	177 (71.1%)	106 (70.2%)	71 (72.4%)	0.8107	0.02	73%	72%	0006
Active smokers	111 (44.6%)	67 (44.4%)	44 (44.9%)	0.9611	0005	45%	45%	0001
Peripheral artery disease	57 (22.9%)	36 (23.8%)	21 (21.4%)	0.7731	0.02	28%	21%	0.06
History of stroke	26 (10.4%)	16 (10.6%)	10 (10.2%)	0.9098	0003	11%	10%	0.008
Chronic kidney disease	65 (26.1%)	40 (26.5%)	25 (25.5%)	0.9806	0.01	32%	26%	0.07
Atrial fibrillation	112 (45%)	60 (39.7%)	52 (53.1%)	0.0530	0.13	52%	53%	0.01
Chronic respiratory disease	30 (25.4%)	29 (19.2%)	24 (24.5%)	0.4027	0.05	21%	24%	0.04
History of ES	49 (19.7%)	20 (13.2%)	29 (29.6%)	**0**.**0026**	0.1635	18%	19%	0.017
History of VT ablation	39 (15.6%)	20 (13.2%)	19 (19.4%)	0.2608	0.0731	16%	15%	−0.0148
Underlying cardiomyopathy								
Ischaemic cardiomyopathy	224 (90%)	134 (88.7%)	90 (91.8%)	0.5632	0.03	88%	92%	0.04
Non-ischaemic cardiomyopathy	25 (10%)	17 (11.3%)	8 (8.2%)	0.5362	0.03	12%	8%	0.04
LVEF (%)	22 [20; 25]	20 [17; 30]	25 [20; 35]	**0.0257**	0.23	25.9 ± 11.8	24.9 ± 11.8	0.02
NYHA III–IV	127 (55%)	83 (55%)	44 (44.9%)	0.1547	0.10	57%	45%	0.011
Baseline treatments								
Amiodarone at baseline	84 (33.7%)	44 (29.1%)	40 (40.8%)	0.0773	0.12	35%	41%	0.005
Betablocker at baseline	204 (83.6%)	118 (80.3%)	86 (88.7%)	0.1199	0.09	82%	80%	0.06
ACE inhibitor	123 (50.4%)	74 (50.3%)	49 (50.5%)	0.9172	0.009	50%	50%	0.0003
ARB	26 (10.7%)	14 (9.5%)	12 (12.4%)	0.6217	0.085	13%	13%	0.078
Aldosterone antagonist	97 (39%)	53 (35.1%)	44 (44.9%)	0.1568	0.09	36%	45%	0.09
ICD	175 (70.3%)	79 (80.6%)	96 (63.6%)	**0.0063**	0.17	79%	81%	0.01
CRT	65 (26.1%)	41 (27.2%)	24 (24.5%)	0.7492	−0.061	22%	35%	0.292
Admission parameters								
Acute pulmonary oedema or cardiogenic shock	65 (26.1%)	53 (35.1%)	12 (12.2%)	**<0.0001**	−0.24	14%	13%	−0.01
Creatinine (µg/dL)	123 [119; 132]	138 [106; 167]	122 [97; 150]	0.1180	−0.24	145.5 ± 64.2	128.2 ± 53.8	−0.28
Management of ES								
Amiodarone in OR	215 (86.3%)	132 (88%)	83 (84.7%)	0.5767	−0.03	82%	85%	0.03
Betablocker in OR	88 (75.2%)	48 (31.7%)	40 (40.8%)	0.8669	0.09	34%	41%	0.06
Cathecholamines	85 (34.1%)	62 (41.1%)	23 (23.5%)	**0.0065**	−0.18	25%	23%	−0.01
Dialysis	12 (4.8%)	10 (6.6%)	2 (2%)	0.1782	−0.05	4%	2%	−0.02
Deep sedation	72 (28.9%)	44 (29.1%)	28 (28.6%)	0.9629	−0.005	19%	29%	0.09
Stellate ganglion block	3 (1.2%)	2 (1.3%)	1 (1%)	0.6950	−0.003	0%	1%	0.005

Continuous quantitative variables with a non-normal distribution are presented as median [interquartile range]. Categorical variables are expressed as the number of patients (percentage). Inverse probability of treatment weighting (IPTW) models were based on propensity scores derived predefined variables (age, NYHA class, acute pulmonary oedema or cardiogenic shock, history of electrical storm, LVEF, catecholamine use, prior ICD implantation, and atrial fibrillation). Standardized mean differences (SMD) are reported before and after IPTW. A post-IPTW SMD below 0.1 was considered indicative of acceptable covariate balance. Weighted means ± SD and proportions were calculated using inverse-probability-of-treatment weights truncated at the 1st and 99th percentiles, with estimates derived via the R survey package. Bold values indicate statistically significant *P*-values (*P* < 0.05).

ACE inhibitor, angiotensin-converting enzyme inhibitor; ARB, angiotensin II receptor blocker; BMI, body mass index; CRT, cardiac resynchronization therapy; ES, electrical storm; VT, ventricular tachycardia; ICD, implantable cardioverter-defibrillator; LVEF, left ventricular ejection fraction.

**Table 3:** Baseline characteristics according to VT ablation in high-risk patients based on the iVT score

Variable	Overall population (*n* = 239)	No ablation (*n* = 154)	Ablation (*n* = 85)	*P*-value	SMD (pre-IPTW)	No ablation IPTW	Ablation IPTW	SMD (post-IPTW)
Male gender	209 (87.4%)	135 (87.6%)	74 (87%)	0.9449	−0.006	88%	84%	−0.02
Age, years	67 [65; 68]	66 [57; 76]	68 [58; 71]	0.5386	0.099	65.5 ± 13	65 ± 12	0.027
BMI (kg/m²)	26.4 [25.7; 27.1]	25.7 [23.4; 29.4]	27.4 [24.4; 29.5]	0.1194	0.11	26.8 ± 5	26.6 ± 4.5	−0.03
Associated comorbidities								
Hypertension	137 (57.3%)	85 (55.2%)	52 (61.2%)	0.4482	0.06	54%	59%	0.09
Diabetes	73 (30.5%)	52 (33.8%)	21 (24.7%)	0.1905	−0.09	33%	25%	−0.08
Dyslipidaemia	143 (59.8%)	91 (29.1%)	52 (61.2%)	0.8595	0.02	59%	58%	−0.008
Active smoking	102 (42.7%)	71 (46.1%)	31 (36.5%)	0.1920	−0.09	48%	35%	−0.13
Peripheral artery disease	38 (15.9%)	23 (14.9%)	15 (17.6%)	0.7158	0.03	14%	17%	0.03
History of stroke	32 (13.4%)	20 (13%)	12 (14.1%)	0.9623	0.011	14%	12%	−0.02
Chronic kidney disease	65 (27.2%)	42 (27.3%)	23 (27.1%)	0.9074	−0.002	29%	22%	−0.07
Atrial fibrillation	101 (42.3%)	61 (39.6%)	40 (47.1%)	0.3275	0.07	43%	45%	0.006
Chronic respiratory disease	31 (13%)	20 (13%)	11 (12.9%)	0.8485	−0.005	12%	10%	−0.02
History of ES	47 (19.7%)	23 (14.9%)	24 (28.2%)	**0**.**0211**	0.1330	19%	20%	0.0498
History of VT ablation	27 (11.2%)	14 (9.1%)	13 (15.3%)	0.2162	0.0422	9%	10%	−0.0236
Underlying cardiomyopathy								
Ischaemic cardiomyopathy	171 (71.5%)	104 (67.5%)	67 (78.8%)	0.0887	0.11	66%	75%	0.09
Non-ischaemic cardiomyopathy	68 (28.5%)	50 (32.5%)	18 (21.2%)		0.11	34%	25%	0.09
LVEF (%)	20 [19.3; 20.8]	20 [15; 25]	20 [20; 25]	0.0722	0.22	20 ± 6	19 ± 6	−0.096
NYHA III–IV	94 (42.9%)	68 (44%)	26 (30.5%)	0.0552	−0.14	40%	44%	0.039
Baseline treatments								
Amiodarone at baseline	74 (31%)	43 (27.9%)	31 (36.5%)	0.2216	0.09	30%	31%	0.005
Betablocker at baseline	192 (81.7%)	119 (78.3%)	73 (88%)	0.0980	0.086	80%	72%	−0.079
ACE inhibitor	113 (48.1%)	70 (46.1%)	43 (51.8%)	0.4794	0.05	46%	51%	0.046
ARB	23 (9.8%)	13 (8.6%)	10 (12%)	0.5272	0.033	9%	11%	0.028
Aldosterone antagonist	98 (41.2%)	55 (35.7%)	43 (51.2%)	**0.0292**	0.15	37%	39%	0.016
ICD	167 (69.9%)	96 (62.3%)	71 (83.5%)	**0.0011**	0.21	69%	65%	−0.04
CRT	64 (26.8%)	43 (27.9%)	21 (24.7%)	0.7003	−0.027	18%	14%	−0.07
Admission parameters								
Acute pulmonary oedema or cardiogenic shock	70 (29.3%)	57 (37%)	13 (15.3%)	**0.0007**	−0.21	30%	33%	0.03
Creatinine (µg/dL)	123 [114; 132]	123 [98; 159]	122 [88; 156]	0.2492	−0.21	140 ± 68	126 ± 51	−0.23
Management of ES								
Amiodarone in OR	205 (85.7%)	131 (85.6%)	74 (87.1%)	0.9109	0.02	84%	89%	0.05
Betablocker in OR	74 (75.5%)	45 (29.2%)	29 (34.1%)	0.5213	0.05	31%	30%	−0.004
Cathecholamines	85 (35.6%)	63 (40.9%)	22 (25.9%)	**0.0291**	−0.15	42%	49%	0.055
Dialysis	17 (7.1%)	15 (9.7%)	2 (2.4%)	0.0623	−0.07	2%	2%	−0.028
Deep sedation	69 (28.9%)	46 (29.9%)	23 (27.1%)	0.7565	−0.028	27%	37%	0.11
Stellate ganglion block	3 (1.3%)	3 (2%)	0 (0%)	0.4938	−0.019	1%	0%	−0.018

Continuous quantitative variables with a non-normal distribution are presented as median [interquartile range]. Categorical variables are expressed as the number of patients (percentage). Inverse probability of treatment weighting (IPTW) models were based on propensity scores derived predefined variables (age, NYHA class, acute pulmonary oedema or cardiogenic shock, history of electrical storm, LVEF, catecholamine use, prior ICD implantation, and atrial fibrillation). Standardized mean differences (SMD) are reported before and after IPTW. A post-IPTW SMD below 0.1 was considered indicative of acceptable covariate balance. Weighted means ± SD and proportions were calculated using inverse-probability-of-treatment weights truncated at the 1st and 99th percentiles, with estimates derived via the R survey package. Bold values indicate statistically significant *P*-values (*P* < 0.05).

ACE inhibitor, angiotensin-converting enzyme inhibitor; ARB, angiotensin II receptor blocker; BMI; body mass index; CRT, cardiac resynchronization therapy; ES, electrical storm; VT, ventricular tachycardia; ICD, implantable cardioverter-defibrillator; LVEF, left ventricular ejection fraction.

Forty-three per cent of lo-PAINESD patients and 47% of the lo-iVT patients underwent VT ablation. Among low-risk patients, those who received VT ablation were more often men, had a higher rate of prior VT ablation, and were more likely to receive beta blockers, while they were also less likely to require catecholamine treatment. Lo-iVT patients who underwent VT ablation were also younger and higher rate of amiodarone treatment prior to admission for ES (see Supplementary material online, *Tables S4* and *S5*).

### Impact of VT ablation on 1-year mortality across risk profiles

The 1-year mortality was 25.5% in the entire cohort. In high-risk patients, it reached 43% in the hi-PAINESD group and 41% in the hi-iVT group, while in low-risk patients, it was 16% (lo-PAINESD) and 18% (lo-iVT). To mitigate confounding factors, we used IPTW based on propensity scores derived from clinically relevant variables—age, NYHA class, acute pulmonary oedema or cardiogenic shock, history of electrical storm, LVEF, catecholamine use, prior ICD implantation, and atrial fibrillation. After weighting, baseline characteristics were well balanced between groups, with SMD below 0.1 in the four subgroups for most variables (*Table 2* for hi-PAINESD, *Table 3* for hi-iVT, Supplementary material online, *Table S4* for lo-PAINESD, and Supplementary material online, *Table S5* for lo-iVT).

In high-risk patients, lower 1-year mortality was observed in those who underwent VT ablation compared with those who did not (*Table 4*), in both adjusted Cox models (hi-PAINESD: 1-year mortality of 54% in patients without ablation vs. 26% with ablation, HR 0.42 [95% CI: 0.26–0.66], *P* = 0.0002; hi-iVT: 1-year mortality of 52% in patients without ablation vs. 20% with ablation, HR 0.31 [0.18–0.53], *P* < 0.0001 *Table 5*) and in IPTW-weighted models (hi-PAINESD: HR 0.54 [0.31–0.93]; hi-iVT: HR 0.39 [0.21–0.74]).

**Table 4:** Cox model for 1-year mortality outcome

	Univariate OR (CI 95%)	Unadjusted *P*-value	Multivariate HR (CI 95%)	Adjusted *P*-value
Overall population				
VT ablation	0.34 (0.24–0.50)	<0.0001	0.41 (0.28–0.59)	<0.0001
History of ES	NR	NR	NR	NR
Ischaemic cardiomyopathy	NR	NR	NR	NR
Use of catecholamines	3.89 (2.85–5.30)	<0.0001	3.47 (2.53–4.75)	<0.0001
High-risk population				
PAINESD				
VT ablation	0.36 (0.23–0.57)	<0.0001	0.42 (0.26–0.66)	0.0002
History of ES	NR	NR	NR	NR
Ischaemic cardiomyopathy	NR	NR	NR	NR
Use of catecholamines	3.27 (2.23–4.80)	<0.0001	2.91 (1.98–4.29)	<0.0001
iVT				
VT ablation	0.29 (0.17–0.49)	<0.0001	0.31 (0.18–0.53)	<0.0001
History of ES	NR	NR	NR	NR
Ischaemic cardiomyopathy	NR	NR	NR	NR
Use of catecholamines	2.68 (1.79–3.99)	<0.0001	2.45 (1.64–3.66)	<0.0001
Low-risk population				
PAINESD				
VT ablation	0.31 (0.16–0.60)	0.0005	0.35 (0.18–0.67)	0.0017
History of ES	NR	NR	NR	NR
Ischaemic cardiomyopathy	0.30 (0.16–0.55)	0.0001	0.31 (0.16–0.57)	0.0002
Use of catecholamines	3.14 (1.79–5.50)	0.0001	2.83 (1.61–4.98)	0.0003
iVT				
VT ablation	0.47 (0.28–0.80)	0.0055	0.57 (0.33–0.98)	0.0436
History of ES	NR	NR	NR	NR
Ischaemic cardiomyopathy	NR	NR	NR	NR
Use of catecholamines	4.43 (2.68–7.32)	<0.0001	3.96 (2.38–6.60)	<0.0001

Hazard ratios (HR) for 1-year mortality associated with VT ablation, derived from a Cox model. Multivariable adjustment included prior history of ES, cardiomyopathy subtype, catecholamine use, and VT ablation as covariates. NR indicates variables not retained in the model.

**Table 5:** Inverse probability of treatment weighting-derived Cox model for 1-year mortality outcome

	HR (CI 95%)	Adjusted *P*-value
High-risk population		
PAINESD		
Adjusted Cox model	0.42 (0.26–0.66)	0.0002
IPTW-derived Cox model	0.54 (0.31–0.93)	0.026
iVT		
Adjusted Cox model	0.31 (0.18–0.53)	<0.0001
IPTW-derived Cox model	0.39 (0.21–0.74)	0.004
Low-risk population		
PAINESD		
Adjusted Cox model	0.35 (0.18–0.67)	0.0017
IPTW-derived Cox model	0.39 (0.19–0.81)	0.011
iVT		
Adjusted Cox model	0.57 (0.33–0.98)	0.0436
IPTW-derived Cox model	0.58 (0.33–0.99)	0.048

Hazard ratios (HR) for 1-year mortality associated with VT ablation, derived from conventional multivariable-adjusted Cox models and IPTW-weighted Cox models. Analyses were performed separately in high-risk and low-risk populations, stratified by PAINESD score and iVT risk score. IPTW models were based on propensity scores derived predefined variables (age, NYHA class, acute pulmonary oedema or cardiogenic shock, history of electrical storm, LVEF, catecholamine use, prior ICD implantation, and atrial fibrillation).

**Figure 3:**
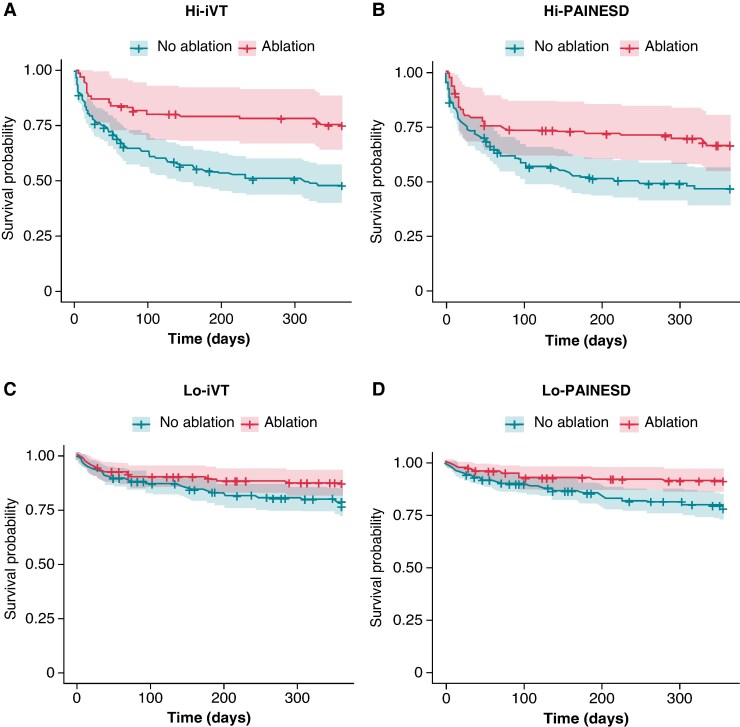
Weighted 1-year survival according to CA-based management in high-risk patients, as defined by PAINESD and iVT scores based on IPTW. IPTW-derived weighted Kaplan–Meier curves for 1-year mortality according to CA-based management for Hi-iVT (*A*), Hi-PAINESD (*B*), Lo-iVT (*C*), and Lo-PAINESD (*D*).

**Figure 4:**
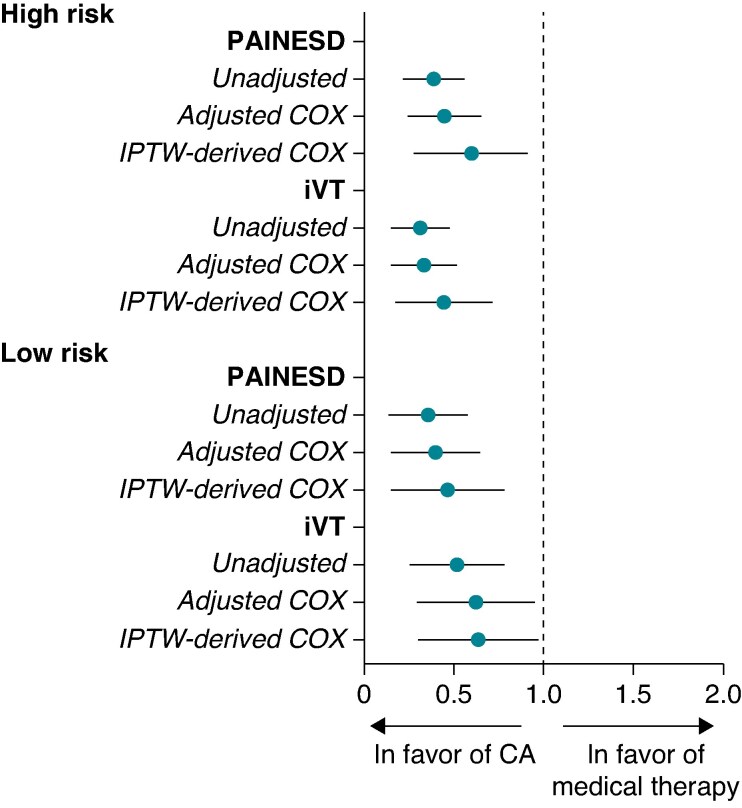
Impact of VT ablation on 1-year mortality across risk profiles. Forest plots illustrating the impact of catheter ablation on 1-year mortality after electrical storm in the overall population and among low- and high-risk patients as assessed by the PAINESD and iVT scores. Adjusted multivariable Cox models included prior history of ES, cardiomyopathy subtype, and the use of catecholamines as competing variables. Inverse probability of treatment weighting (IPTW) models were based on propensity scores derived predefined variables (age, NYHA class, acute pulmonary oedema or cardiogenic shock, history of electrical storm, LVEF, catecholamine use, prior ICD implantation, and atrial fibrillation).

Among low-risk patients, lower 1-year mortality was observed in those who underwent VT ablation compared with those who did not (lo-PAINESD: 21% mortality without ablation vs. 8% with ablation, adjusted Cox model: HR 0.35 [95% CI: 0.18–0.67], *P* = 0.0017; lo-iVT: 22% without ablation vs. 11% with ablation, adjusted Cox model: HR 0.57 [0.33–0.98], *P* = 0.0436 *Table 5*). These findings were consistent in IPTW-weighted models (lo-PAINESD: HR 0.39 [0.19–0.82]; lo-iVT: HR 0.58 [0.33–0.99]). These results were consistently supported by Kaplan–Meier survival curves in both unadjusted and IPTW-weighted analyses (*Figure 3* and Supplementary material online, *Figure S1*), which confirmed the association between VT ablation and improved survival in both high- and low-risk patients. *Figure 4* displays a forest plot illustrating the association between VT ablation and 1-year mortality across risk groups, stratified by the statistical model used.

**Figure 5:**
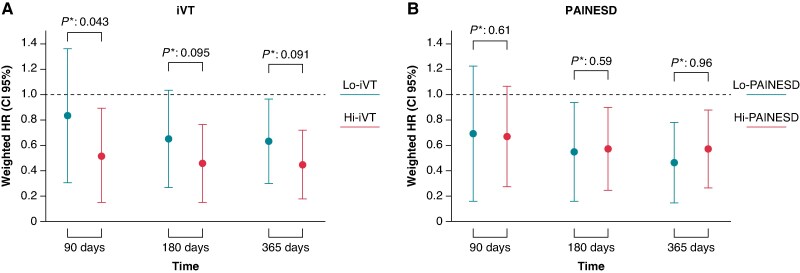
Time-dependent effect of ventricular tachycardia (VT) ablation on mortality, weighted by IPTW, stratified by iVT and PAINESD risk scores. Panels show weighted hazard ratios (HRs) and 95% confidence intervals for all-cause mortality at 90, 180, and 365 days following electrical storm, estimated by IPTW-adjusted Cox models: (*A*) Stratification by iVT score. (*B*) Stratification by PAINESD score. The horizontal dashed red line indicates HR = 1 (no effect). Brackets above each time point display the *P*-value for the ablation × risk group interaction test at each horizon.

We then formally tested whether the survival benefit of VT ablation differed between low- and high-risk patients by fitting IPTW-weighted Cox models with an ablation × risk-group interaction at 90, 180, and 365 days. In IPTW-weighted interaction analyses, stratification by the iVT score—but not by PAINESD—identified a subgroup of high-risk patients who derive a significantly earlier survival benefit from VT ablation, with the interaction reaching significance at 90 days (*P* = 0.043) and trending at 180 days (*P* = 0.095) and 365 days (*P* = 0.091) (*Figure 5A*). In contrast, stratification by PAINESD yielded no significant interaction at any time-point (*P* = 0.61, 0.59, and 0.96 for 90, 180, and 365 days, respectively), demonstrating that the timing and magnitude of benefit were similar in low- and high-PAINESD patients (*Figure 5B*). Taken together, these findings raise the possibility that the iVT score could help identify high-risk patients who may experience a more rapid mortality reduction following VT ablation.

## Discussion

To the best of our knowledge, this study is the first to report the impact of CA of ES in different level of risk profile. The main findings are: (i) a significant proportion of high-risk patients among patients presenting ES—41% based on the PAINESD score and 39.4% based on the iVT score, (ii) a rate of VT ablation reaching 42.4% in the overall population, with rates of 39.4% in hi-PAINESD patients and 35.5% in hi-iVT patients, and (iii) lower 1-year mortality associated with CA in high-risk patients.

### ES in structural heart disease

Patients with structural heart disease who present with ES are typically older, more often male, have lower LVEF, advanced HF, and more cardiovascular comorbidities compared to those without ES.^12,13^ These distinct clinical characteristics add considerable complexity to ES management and affect prognosis significantly.^14^ Consequently, ES poses a major mortality risk, especially within the first three months following onset. Additionally, the 1-year mortality rate after hospitalization for ES is alarmingly high at ∼35%,^2^ increasing to 40–45% in patients who do not respond to CA. Our findings are in line with these previous studies, reaching 12% at 1 month and 26% at one year.^15^

### VT ablation in the setting of ES

ES represents a high-risk scenario for patients undergoing VT ablation. In a study of 2061 patients who underwent VT ablation, Santangeli *et al*.^6^ reported that patients with ES faced a 3.61-fold higher risk of early mortality post-ablation. Additionally, a 5.12-fold increase in the risk of acute haemodynamic decompensation was previously reported in this context.^4^ While no randomized trials have yet evaluated the benefits of VT ablation specifically for ES, observational studies have suggested that ablation in this setting may be associated with reduced mortality.^16^ Previous results from this cohort demonstrated a significant benefit from VT ablation, with a 59% lower 1-year mortality using propensity-matched analysis.^7^ However, the current definition of ES includes a broad range of clinical situations, reflecting varying VT burdens and risk profiles based on underlying conditions, raising questions about the effect of ablation across different risk profiles.

### VT ablation in high-risk profiles

VT ablation is a key strategy for managing drug-refractory VT-ES, though it carries a substantial risk of periprocedural complications and adverse outcomes.^17^ A particular concern is periprocedural AHD, which is closely linked to higher short-term mortality following the procedure. Consequently, there is increasing interest in identifying patients at high risk for adverse outcomes after VT ablation.

Our study adds further evidence to the potential benefits of VT ablation in patients with ES across various risk profiles. In this study, which focused exclusively on ES patients, we found that nearly 40% fell into high-risk categories based on PAINESD and iVT scores—a previously unreported proportion. Compared to the original study defining the iVT score, where about one-third of patients had a high risk of postoperative mortality, this higher proportion may reflect the greater comorbidity burden in the ES population relative to a broader VT ablation group. However, high-risk patients in the iVT cohort showed a 1-year mortality risk of up to 20%, consistent with the rates observed in our high-risk (hi-iVT) group undergoing VT ablation.

Notably, we found that high-risk patients who underwent VT ablation had lower mortality rates than those who did not undergo the procedure. The lower occurrence of mortality was evident across both low- and high-risk profiles, though with different timelines In time-stratified IPTW-weighted Cox analyses, VT ablation was linked to a survival benefit in hi-iVT patients as early as 90 days (interaction *P* = 0.043), which persisted at 6 months and 1 year, whereas among low-risk patients a statistically significant association emerged only at 1 year. This clear temporal heterogeneity indicates that high-risk individuals derive the most rapid mortality reduction from ablation, highlighting the need to prioritize and expedite the procedure in this subgroup following an electrical storm. At the same time, these findings must be interpreted against the backdrop of current European practice. The 2023 EHRA survey revealed substantial variability across Europe in the use and timing of VT ablation and adjunctive therapies.^18^ Such heterogeneity highlights the need for more standardized management pathways to ensure that high-risk iVT patients can access timely ablation and realize the early survival benefits suggested here.

While our data emphasize the need to streamline access to VT ablation for high-risk iVT patients, this strategy must be balanced against the procedure’s inherent risks. The rate of ischaemic stroke rate observed in our cohort (2.7%) was higher than previously reported in VT ablation series performed in elective settings, where rates typically range between 0.5% and 1.0%. This higher ischaemic stroke rate may be explained by the clinical severity of our population, characterized by frequent haemodynamic instability and emergent procedures in the context of electrical storm. These factors may have limited the possibility of optimal periprocedural anticoagulation management. Furthermore, the inclusion of patients referred to tertiary centres for complex or high-risk procedures likely contributed to an overestimation of complication rates compared with elective ablation series. Our findings are consistent with previous reports indicating increased procedural risk in unstable patients undergoing VT ablation.^6^ The timing of VT ablation procedures (emergency vs. elective, weekdays vs. weekends) was not systematically recorded in this cohort, thus limiting our ability to assess potential differences in outcomes related to procedural timing. Recent literature indicates that emergency or out-of-hours VT ablation procedures might be associated with different outcomes due to logistical, resource-based, or clinical severity factors.^19^ Future studies should explore how these factors might influence patient outcomes in the context of electrical storm.

### Study limitations

This study has several limitations. As an observational study conducted across four tertiary arrhythmia centres, patient characteristics and outcomes may be influenced by referral bias, though they are representative of patients admitted for ES in similar institutions. CA for ES is frequently performed in high-volume ablation centres with expertise in ventricular arrhythmia ablation and access to advanced heart failure support. The relative low rate of adverse events reported in this population may not be representative of larger scale registries. Despite these limitations, this study provides, for the first time, a comprehensive overview of the impact of CA for ES on survival outcomes in ES patients at high risk for postoperative mortality. Furthermore, the consistency of our findings across both multivariable-adjusted Cox models and IPTW-weighted survival analyses further reinforces the robustness of the observed association between VT ablation and improved survival. This convergence of analytical approaches supports the validity of our results despite the inherent limitations of observational data. However, even with these adjustments, residual confounding—particularly related to unmeasured variables and selection bias—cannot be excluded. As such, our findings should be interpreted with caution and considered hypothesis-generating rather than definitive evidence of a causal relationship.

## Conclusion

In this French multicentre population of patients experiencing electrical storm, 41% were considered as high risk of acute haemodynamic decompensation and mortality according to the PAINESD score, and 39.4% according to the iVT score. VT ablation was associated with a lower 1-year mortality in low- and high-risk patients, with a clinical benefit appearing earlier in high-risk patients.

## Supplementary Material

euaf188_Supplementary_Data

## Data Availability

Data supporting the findings of this study are available from the corresponding author upon reasonable request.
