# Supplementary material for: Impact of ventricular tachycardia ablation in the setting of electrical storm based on patient risk profile
Source: Europace. 2025 Aug 20;27(9):euaf188. doi: 10.1093/europace/euaf188 (PMC12448930; doi:10.1093/europace/euaf188)
Supplement: euaf188_Supplementary_Data [file euaf188_supplementary_data.docx]

**SUPPLEMENTARY MATERIALS**

Table S1. Baseline characteristics according risk profile based on the PAINESD.

|  | Overall population  n = 606 | High risk  n = 249 | Low risk  n = 357 | *p-value* |
| --- | --- | --- | --- | --- |
| Gender (Male) | 520 (85,4%) | 223 (89.6%) | 297 (83.2%) | **0.0365** |
| Age | 67 [59 ; 74] | 70 [68 ; 71] | 65 [63 ; 66.5] | **< 0.0001** |
| BMI (kg/m^2^) | 27.08 [24.22 ; 30.45] | 27.4 [26.7 ; 28] | 26.8 [26.1 ; 27.3] | **0.0469** |
| **Associated Comorbidities** | | | | |
| Hypertension | 337 (55.6%) | 171 (68.7%) | 166 (46.5%) | **< 0.0001** |
| Diabetes | 170 (28.1%) | 130 (52.2%) | 40 (11.2%) | **< 0.0001** |
| Dyslipidemia | 349 (57.6%) | 177 (71.1%) | 172 (48.2%) | **< 0.0001** |
| Active smokers | 247 (40.8%) | 111 (44.6%) | 136 (38.1%) | 0.1300 |
| Peripheral artery desease | 85 (14%) | 57 (22.9%) | 28 (7.8%) | **< 0.0001** |
| History of stoke | 64 (10.6%) | 26 (10.4%) | 38 (10.6%) | 0.9565 |
| Chronic kidney disease | 130 (21.5%) | 65 (26.1%) | 65 (18.2%) | **0.0258** |
| History of atrial fibrillation | 265 (43.7%) | 112 (45%) | 153 (42.8%) | 0.6635 |
| Chronic respiratory disease | 71 (11.7%) | 30 (25.4%) | 18 (5%) | **< 0.0001** |
| History of ES | 109 (18%) | 49 (19.7%) | 60 (16.8%) | 0.4248 |
| History of VT ablation | 89 (14.7%) | 39 (15.6%) | 50 (14%) | 0.6525 |
| **Underlying cardiomyopathy** | | | | |
| Ischemic cardiomyopathy | 393 (64.9%) | 224 (90%) | 169 (47.3%) | **< 0.0001** |
| Non Ischemic cardiomyopathy | 213 (35.1%) | 25 (10%) | 188 (52.6%) | **< 0.0001** |
| LVEF (%) | 30 [20 ; 42] | 22 [20 ; 25] | 35 [35 ; 37.5] | **< 0.0001** |
| NYHA III-IV | 163 (29.5%) | 127 (55%) | 36 (10%) | **< 0.0001** |
| **Baseline treatments** | | | | |
| Amiodarone | 185 (30.6%) | 84 (33.7%) | 101 (28.2%) | 0.1870 |
| Betablockers | 494 (83%) | 204 (83.6%) | 290 (81.2%) | 0.8384 |
| ACE inhibitor | 289 (48.6%) | 123 (50.4%) | 166 (46.4%) | 0.5062 |
| ARB | 60 (10.1%) | 26 (10.7%) | 34 (9.5%) | 0.8043 |
| Aldosterone antagonist | 195 (32.3%) | 97 (39%) | 98 (27.4%) | 0.0044 |
| ICD | 412 (68%) | 175 (70.3%) | 237 (66.4%) | 0.3562 |
| CRT | 135 (22,3%) | 65 (26.1%) | 70 (19.6%) | 0.0732 |
| **Admission parameters** | | | | |
| Acute pulmonary oedema or Cardiogenic shock | 99 (16.3%) | 65 (26.1%) | 34 (9.5%) | **< 0.0001** |
| Creatinin (ug/dL) | 114 [88 ; 150] | 123 [119 ; 132] | 104 [97.2 ; 106] | **< 0.0001** |
| **Management of ES** | | | | |
| Amiodarone | 506 (83.6%) | 215 (86.3%) | 291 (81.5%) | 0.1958 |
| Betablockers | 228 (37.6%) | 88 (75.2%) | 140 (39.2%) | 0.7760 |
| Cathecholamines | 142 (23.4%) | 85 (34.1%) | 57 (15.9%) | **< 0.0001** |
| Dialysis | 26 (4.3%) | 12 (4.8%) | 14 (3.9%) | 0.7393 |
| Deep sedation | 138 (22.8%) | 72 (28.9%) | 66 (18.4%) | **0.0036** |
| Stellate ganglion block | 10 (1.7%) | 3 (1.2%) | 7 (1.9%) | 0.7037 |

Continuous quantitative variables with a non-normal distribution are presented as median [interquartile range]. Categorical variables are expressed as the number of patients (percentage). Abbreviations: ACE inhibitor : angiotensin-converting enzyme inhibitor, ARB : Angiotensin II Receptor Blocker BMI : Body Mass Index, ; CRT : Cardiac Resynchronization Therapy, ES : Electrical storm, VT : Ventricular Tachycardia, , ICD : implantable cardioverter-defibrillator, LVEF: left ventricular ejection fraction.

Table S2. Baseline characteristics according risk profile based on the iVT score.

|  | Overall population  n = 606 | High risk  n = 239 | Low risk  n = 367 | *p-value* |
| --- | --- | --- | --- | --- |
| Gender (Male) | 520 (85,4%) | 209 (87.4%) | 311 (84.7%) | 0.4156 |
| Age | 67 [59 ; 74] | 67 [65 ; 68] | 67 [66 ; 69] | 0.4181 |
| BMI (kg/m^2^) | 27.08 [24.22 ; 30.45] | 26.4 [25.7 ; 27.1] | 27.4 [26.9 ; 28] | **0.0131** |
| **Associated Comorbidities** | | | | |
| Hypertension | 337 (55.6%) | 137 (57.3%) | 200 (54.5%) | 0.5480 |
| Diabetes | 170 (28.1%) | 73 (30.5%) | 97 (26.4%) | 0.3130 |
| Dyslipidemia | 349 (57.6%) | 143 (59.8%) | 206 (56.1%) | 0.4139 |
| Active smokers | 247 (40.8%) | 102 (42.7%) | 145 (39.5%) | 0.4895 |
| Peripheral artery desease | 85 (14%) | 38 (15.9%) | 47 (12.8%) | 0.3411 |
| History of stoke | 64 (10.6%) | 32 (13.4%) | 32 (8.7%) | 0.0905 |
| Chronic kidney disease | 130 (21.5%) | 65 (27.2%) | 65 (17.7%) | **0.0074** |
| History of atrial fibrillation | 265 (43.7%) | 101 (42.3%) | 164 (44.6%) | 0.6136 |
| Chronic respiratory disease | 71 (11.7%) | 31 (13%) | 40 (10.8%) | 0.5185 |
| History of ES | 109 (18%) | 47 (19.7%) | 62 (16.8%) | 0.4473 |
| History of VT ablation | 89 (14.7%) | 27 (11.2%) | 62 (16.8%) | **0.0743** |
| **Underlying cardiomyopathy** | | | | |
| Ischemic cardiomyopathy | 393 (64.9%) | 171 (71.5%) | 222 (60.4%) | 0.0069 |
| Non Ischemic cardiomyopathy | 213 (35.1%) | 68 (28.5%) | 145 (39.5%) | 0.0069 |
| LVEF (%) | 30 [20 ; 42] | 20 [19.3 ; 20.8] | 40 [37 ; 40] | **< 0.0001** |
| NYHA III-IV | 163 (29.5%) | 94 (42.9%) | 69 (18.8%) | **< 0.0001** |
| **Baseline treatments** | | | | |
| Amiodarone | 185 (30.6%) | 74 (31%) | 111 (30.2%) | 0.9399 |
| Betablockers | 494 (83%) | 192 (81.7%) | 302 (82.2%) | 0.5600 |
| ACE inhibitor | 289 (48.6%) | 113 (48.1%) | 176 (47.9%) | 0.9141 |
| ARB | 60 (10.1%) | 23 (9.8%) | 37 (10%) | 0.9561 |
| Aldosterone antagonist | 195 (32.3%) | 98 (41.2%) | 97 (26.4%) | **0.0002** |
| ICD | 412 (68%) | 167 (69.9%) | 245 (66.8%) | 0.4748 |
| CRT | 135 (22,3%) | 64 (26.8%) | 71 (19.3%) | **0.0405** |
| **Admission parameters** | | | | |
| Acute pulmonary oedema or Cardiogenic shock | 99 (16.3%) | 70 (29.3%) | 29 (7.9%) | **< 0.0001** |
| Creatinin (ug/dL) | 114 [88 ; 150] | 123 [114 ; 132] | 106 [99.1 ; 110] | **< 0.0001** |
| **Management of ES** | | | | |
| Amiodarone | 506 (83.6%) | 205 (85.7%) | 301 (82%) | 0.9074 |
| Betablockers | 228 (37.6%) | 74 (75.5%) | 154 (41.9%) | 0.8890 |
| Cathecholamines | 142 (23.4%) | 85 (35.6%) | 57 (15.5%) | **< 0.0001** |
| Dialysis | 26 (4.3%) | 17 (7.1%) | 9 (2.4%) | **0.0104** |
| Deep sedation | 138 (22.8%) | 69 (28.9%) | 69 (18.8%) | **0.0053** |
| Stellate ganglion block | 10 (1.7%) | 3 (1.3%) | 7 (1.9%) | 0.7755 |

Continuous quantitative variables with a non-normal distribution are presented as median [interquartile range]. Categorical variables are expressed as the number of patients (percentage). Abbreviations: ACE inhibitor : angiotensin-converting enzyme inhibitor, ARB : Angiotensin II Receptor Blocker BMI : Body Mass Index, ; CRT : Cardiac Resynchronization Therapy, ES : Electrical storm, VT : Ventricular Tachycardia, , ICD : implantable cardioverter-defibrillator, LVEF: left ventricular ejection fraction.

Table S3. VT ablation procedural characteristics.

|  | Overall population  n = 257 |
| --- | --- |
| Epicardial ablation | 42 (14.6) |
| Procedural time, min | 186 ± 55 |
| Severe complications | 14 (5.4) |
| Stroke/transient ischemic attack | 7 (2.7) |
| Tamponade | 2 (0.08) |
| Pericardial effusion | 2 (0.08) |
| Arteriovenous fistula | 3 (0.1) |
| Procedure-related death | 0 (0) |

Continuous quantitative variables with a non-normal distribution are presented as median [interquartile range]. Categorical variables are expressed as the number of patients (percentage).

Table S4. Baseline characteristics according to VT ablation in low and intermediate risk patients based on the PAINESD score.

| Variable | Overall population (n = 357) | No Ablation (n = 198) | Ablation (n = 159) | p-value | SMD (pre-IPTW) | No Ablation IPTW | Ablation IPTW | SMD (post-IPTW) |
| --- | --- | --- | --- | --- | --- | --- | --- | --- |
| Male gender | 297 (83.2%) | 155 (78.2%) | 142 (89.3%) | 0.0086 | 0.117287 | 82% | 75% | -0.07123 |
| Age, years | 65 [63 ; 66.5] | 65 [53 ; 75] | 64 [54 ; 87] | 0.2113 | -0.1666 | 63±15 | 64±15 | 0.083404 |
| BMI (kg/m²) | 26.8 [26.1 ; 27.3] | 26.2 [23.1 ; 29.6] | 27.1 [24.6 ; 30.1] | 0.0528 | 0.141845 | 27.3±5.5 | 26.8±4.8 | -0.09755 |
| **Associated comorbidities** | | | | | | | | |
| Hypertension | 166 (46.5%) | 88 (44.4%) | 78 (49.1%) | 0.4463 | 0.032589 | 46% | 53% | 0.07137 |
| Diabetes | 40 (11.2%) | 24 (12.1%) | 16 (10.1%) | 0.6570 | -0.01985 | 12% | 9% | -0.03602 |
| Dyslipidemia | 172 (48.2%) | 95 (48%) | 77 (48.4%) | 0.9821 | 0.00182 | 50% | 49% | -0.01154 |
| Active smoking | 136 (38.1%) | 73 (36.9%) | 63 (39.6%) | 0.6724 | 0.035897 | 40% | 38% | -0.025 |
| Peripheral artery disease | 28 (7.8%) | 14 (7.1%) | 14 (8.8%) | 0.6835 | 0.028784 | 7% | 7% | 0.004247 |
| History of stroke | 38 (10.6%) | 23 (11.6%) | 15 (9.4%) | 0.6223 | -0.02763 | 12% | 9% | -0.03275 |
| Chronic kidney disease | 65 (18.2%) | 42 (21.2%) | 23 (14.5%) | 0.1326 | -0.05674 | 22% | 22% | -0.00361 |
| Atrial fibrillation | 153 (42.8%) | 87 (43.9%) | 66 (41.5%) | 0.7237 | -0.02812 | 45% | 49% | 0.037618 |
| Chronic respiratory disease | 18 (5%) | 11 (5.6%) | 7 (4.4%) | 0.8014 | -0.01125 | 5% | 4% | -0.01628 |
| History of ES | 60 (16.8%) | 30 (15.2%) | 30 (18.9%) | 0.4290 | 0.0372 | 18% | 15% | -0.0351 |
| History of VT ablation | 50 (14%) | 20 (10.1%) | 30 (18.9%) | 0.0265 | 0.0892 | 12% | 16% | 0.0404 |
| **Underlying cardiomyopathy** | | | | | | | | |
| Ischemic cardiopathy | 169 (47.3%) | 87 (43.9%) | 82 (51.6%) | 0.1839 | 0.080232 | 49% | 45% | -0.04673 |
| Non ischemic cardiopathy | 188 (52.6%) | 111 (56.1%) | 77 (48.4%) | 0.1839 | 0.080232 | 51% | 55% | -0.04673 |
| LVEF (%) | 35 [35 ; 37.5] | 35 [25 ; 45] | 35 [30 ; 45] | 0.1647 | 0.1301 | 37.1±13.8 | 35.9±12.3 | -0.08112 |
| NYHA III-IV | 36 (10%) | 24 (12.1%) | 12 (7.5%) | 0.2114 | -0.04566 | 10% | 11% | 0.009217 |
| **Baseline treatments** | | | | | | | | |
| Amiodarone at baseline | 101 (28.2%) | 49 (24.7%) | 52 (32.9%) | 0.1143 | 0.076427 | 29% | 28% | -0.00876 |
| Betablocker at baseline | 290 (81.2%) | 148 (74%) | 142 (91.6%) | 0.0001 | 0.148056 | 79% | 85% | 0.054788 |
| ACE Inhibitor | 166 (46.4%) | 80 (40.8%) | 86 (55.5%) | 0.0087 | 0.148387 | 43% | 49% | 0.068106 |
| ARB | 34 (9.5%) | 21 (10.7%) | 13 (8.4%) | 0.5821 | -0.03027 | 10% | 6% | -0.03793 |
| Aldosterone antagonist | 98 (27.4%) | 46 (23.2%) | 52 (33.1%) | 0.0511 | 0.098263 | 25% | 28% | 0.026879 |
| ICD | 237 (66.4%) | 113 (57.1%) | 124 (78%) | 0.0001 | 0.204963 | 65% | 58% | -0.06871 |
| CRT | 70 (19.6%) | 42 (21.1%) | 28 (17.6%) | 0.4728 | 0.003 | 15% | 18% | -0.036 |
| **Admission parameters** | | | | | | | | |
| Acute pulmonary oedema or cardiogenic shock | 34 (9.5%) | 27 (13.6%) | 7 (4.4%) | 0.0056 | -0.14946 | 11% | 10% | -0.01141 |
| Creatinin (µg/dL) | 104 [97.2 ; 106] | 105 [79 ; 142] | 101 [83 ; 127] | 0.6524 | -0.17172 | 121±73 | 118±47 | -0.04973 |
| **Management of ES** | | | | | | | | |
| Amiodarone in OR | 291 (81.5%) | 162 (81.8%) | 129 (81.6%) | 0.9234 | -0.00893 | 83% | 84% | 0.007248 |
| Betablocker in OR | 140 (39.2%) | 60 (30.3%) | 80 (50.3%) | 0.1113 | 0.212242 | 49% | 21% | 0.160587 |
| Cathecholamines | 57 (15.9%) | 40 (20.2%) | 17 (10.7%) | 0.0219 | -0.09545 | 18% | 13% | -0.04701 |
| Dialysis | 14 (3.9%) | 10 (5.1%) | 4 (2.5%) | 0.3411 | -0.02548 | 4% | 3% | -0.01189 |
| Deep sedation | 66 (18.4%) | 37 (18.7%) | 29 (18.2%) | 0.9770 | 0.002481 | 17% | 22% | 0.042824 |
| Stellate ganglion block | 7 (1.9%) | 3 (1.5%) | 4 (2.5%) | 0.7625 | 0.010422 | 3% | 1% | 0.013629 |

Continuous quantitative variables with a non-normal distribution are presented as median [interquartile range]. Categorical variables are expressed as the number of patients (percentage). Abbreviations: ACE inhibitor : angiotensin-converting enzyme inhibitor, ARB : Angiotensin II Receptor Blocker BMI : Body Mass Index, ; CRT : Cardiac Resynchronization Therapy, ES : Electrical storm, VT : Ventricular Tachycardia, , ICD : implantable cardioverter-defibrillator, LVEF: left ventricular ejection fraction. Inverse probability of treatment weighting (IPTW) models were based on propensity scores derived predefined variables (age, NYHA class, acute pulmonary edema or cardiogenic shock, history of electrical storm, LVEF, catecholamine use, prior ICD implantation, and atrial fibrillation). Standardized mean differences (SMD) are reported before and after IPTW. A post-IPTW SMD below 0.1 was considered indicative of acceptable covariate balance. Weighted means ± SD and proportions were calculated using inverse‑probability‑of‑treatment weights truncated at the 1st and 99th percentiles, with estimates derived via the R survey package.

Table S5. Baseline characteristics according to VT ablation in low risk patients based on the iVT score.

| Variable | Overall population (n = 367) | No Ablation (n = 195) | Ablation (n = 172) | p-value | SMD (pre-IPTW) | No Ablation IPTW | Ablation IPTW | SMD (post-IPTW) |
| --- | --- | --- | --- | --- | --- | --- | --- | --- |
| Male gender | 311 (84.7%) | 154 (78.9%) | 157 (91.2%) | 0.0018 | 0.301 | 81.4% | 82.6% | 0.030 |
| Age, years | 67 [66 ; 69] | 70 [59 ; 76] | 67 [59 ; 73] | 0.0176 | -0.222 | 66.3±12 | 66.2±12 | -0.025 |
| BMI (kg/m²) | 27.4 [26.9 ; 28] | 27 [24.1 ; 30.4] | 27.9 [24.8 ; 31.1] | 0.1391 | 0.142 | 27.8±4.3 | 28.1±4.2 | -0.007 |
| **Associated comorbidities** | | | | | | | | |
| Hypertension | 200 (54.5%) | 103 (52.8%) | 97 (56.4%) | 0.5611 | 0.070 | 52% | 59% | 0.069 |
| Diabetes | 97 (26.4%) | 55 (28.2%) | 42 (24.4%) | 0.4825 | -0.085 | 25% | 24% | -0.015 |
| Dyslipidemia | 206 (56.1%) | 110 (56.4%) | 96 (55.8%) | 0.9924 | -0.013 | 55% | 56% | -0.004 |
| Tabagism | 145 (39.5%) | 69 (35.4%) | 76 (44.2%) | 0.1065 | 0.179 | 38% | 38% | 0.012 |
| Peripheral artery disease | 47 (12.8%) | 27 (13.8%) | 20 (11.6%) | 0.6326 | -0.063 | 12% | 11% | -0.046 |
| History of stroke | 32 (8.7%) | 19 (9.7%) | 13 (7.6%) | 0.5788 | -0.074 | 8% | 8% | -0.016 |
| Chronic kidney disease | 65 (17.7%) | 40 (20.5%) | 25 (14.5%) | 0.1738 | -0.161 | 17% | 17% | -0.021 |
| Atrial fibrillation | 164 (44.6%) | 86 (44.1%) | 78 (45.3%) | 0.8931 | 0.021 | 43% | 44% | -0.014 |
| Chronic respiratory disease | 40 (10.8%) | 20 (10.3%) | 20 (11.6%) | 0.8003 | 0.038 | 9% | 10% | -0.040 |
| History of ES | 62 (16.8%) | 27 (13.8%) | 35 (20.3%) | 0.1286 | 0.0650 | 17% | 17% | -0.0095 |
| History of VT ablation | 62 (16.8%) | 26 (13.3%) | 36 (20.9%) | 0.0721 | 0.1036 | 14% | 16% | 0.0439 |
| **Underlying cardiomyopathy** | | | | | | | | |
| Ischemic cardiopathy | 222 (60.4%) | 117 (60%) | 105 (61%) | 0.9222 | 0.021 | 60% | 62% | 0.012 |
| Non ischemic cardiopathy | 145 (39.5%) | 78 (40%) | 67 (39%) | 0.9222 | 0.021 | 40% | 38% | 0.012 |
| LVEF (%) | 40 [37 ; 40] | 40 [30 ; 72] | 40 [34 ; 45] | 0.9540 | -0.022 | 40.0±11 | 40.0±10 | -0.007 |
| NYHA III-IV | 69 (18.8%) | 39 (20%) | 30 (17.4%) | 0.6227 | -0.069 | 17% | 16% | -0.039 |
| **Baseline treatments** | | | | | | | | |
| Amiodarone at baseline | 111 (30.2%) | 50 (25.6%) | 61 (35.7%) | 0.0489 | 0.220 | 29% | 30% | -0.002 |
| Betablocker at baseline | 302 (82.2%) | 147 (77%) | 155 (91.7%) | 0.0003 | 0.389 | 78% | 80% | 0.000 |
| ACE Inhibitor | 176 (47.9%) | 84 (44%) | 92 (54.4%) | 0.0607 | 0.211 | 46% | 47% | 0.010 |
| ARB | 37 (10%) | 22 (11.5%) | 15 (8.9%) | 0.5156 | -0.085 | 10% | 9% | -0.028 |
| Aldosterone antagonist | 97 (26.4%) | 44 (22.6%) | 53 (31%) | 0.0883 | 0.185 | 25% | 26% | 0.003 |
| ICD | 245 (66.8%) | 113 (57.9%) | 132 (76.7%) | 0.0002 | -0.393 | 60% | 64% | 0.061 |
| CRT | 71 (19.3%) | 40 (20.5%) | 31 (18%) | 0.6383 | -0.066 | 18% | 22% | 0.087 |
| **Admission parameters** | | | | | | | | |
| Acute pulmonary oedema or cardiogenic shock | 29 (7.9%) | 23 (11.8%) | 6 (3.5%) | 0.0060 | -0.325 | 7% | 6% | -0.049 |
| Creatinin (µg/dL) | 106 [99.1 ; 110] | 106 [79 ; 144] | 105 [85 ; 125] | 0.7416 | -0.017 | 126.0±69 | 113.0±50 | -0.089 |
| **Management of ES** | | | | | | | | |
| Amiodarone in OR | 301 (82%) | 163 (83.6%) | 138 (80.7%) | 0.5591 | -0.070 | 82% | 82% | -0.013 |
| Betablocker in OR | 154 (41.9%) | 63 (32.3%) | 91 (52.9%) | 0.2369 | 0.224 | 35% | 37% | -0.004 |
| Cathecholamines | 57 (15.5%) | 39 (20%) | 18 (10.5%) | 0.0177 | -0.230 | 19% | 12% | -0.165 |
| Dialysis | 9 (2.4%) | 5 (2.6%) | 4 (2.3%) | 0.8487 | -0.012 | 2% | 2% | -0.024 |
| Deep sedation | 69 (18.8%) | 35 (17.9%) | 34 (19.8%) | 0.7557 | 0.051 | 15% | 25% | 0.157 |
| Stellate ganglion block | 7 (1.9%) | 2 (1%) | 5 (2.9%) | 0.3429 | 0.090 | 1% | 1% | -0.029 |

Continuous quantitative variables with a non-normal distribution are presented as median [interquartile range]. Categorical variables are expressed as the number of patients (percentage). Abbreviations: ACE inhibitor : angiotensin-converting enzyme inhibitor, ARB : Angiotensin II Receptor Blocker BMI : Body Mass Index, ; CRT : Cardiac Resynchronization Therapy, ES : Electrical storm, VT : Ventricular Tachycardia, , ICD : implantable cardioverter-defibrillator, LVEF: left ventricular ejection fraction. Inverse probability of treatment weighting (IPTW) models were based on propensity scores derived predefined variables(age, NYHA class, acute pulmonary edema or cardiogenic shock, history of electrical storm, LVEF, catecholamine use, prior ICD implantation, and atrial fibrillation) .Standardized mean differences (SMD) are reported before and after IPTW. A post-IPTW SMD below 0.1 was considered indicative of acceptable covariate balance. Weighted means ± SD and proportions were calculated using inverse‑probability‑of‑treatment weights truncated at the 1st and 99th percentiles, with estimates derived via the R survey package.

**Figure S1. Unweighted 1-year survival according to CA-based management across risk profiles, as defined by PAINESD and iVT scores**

**
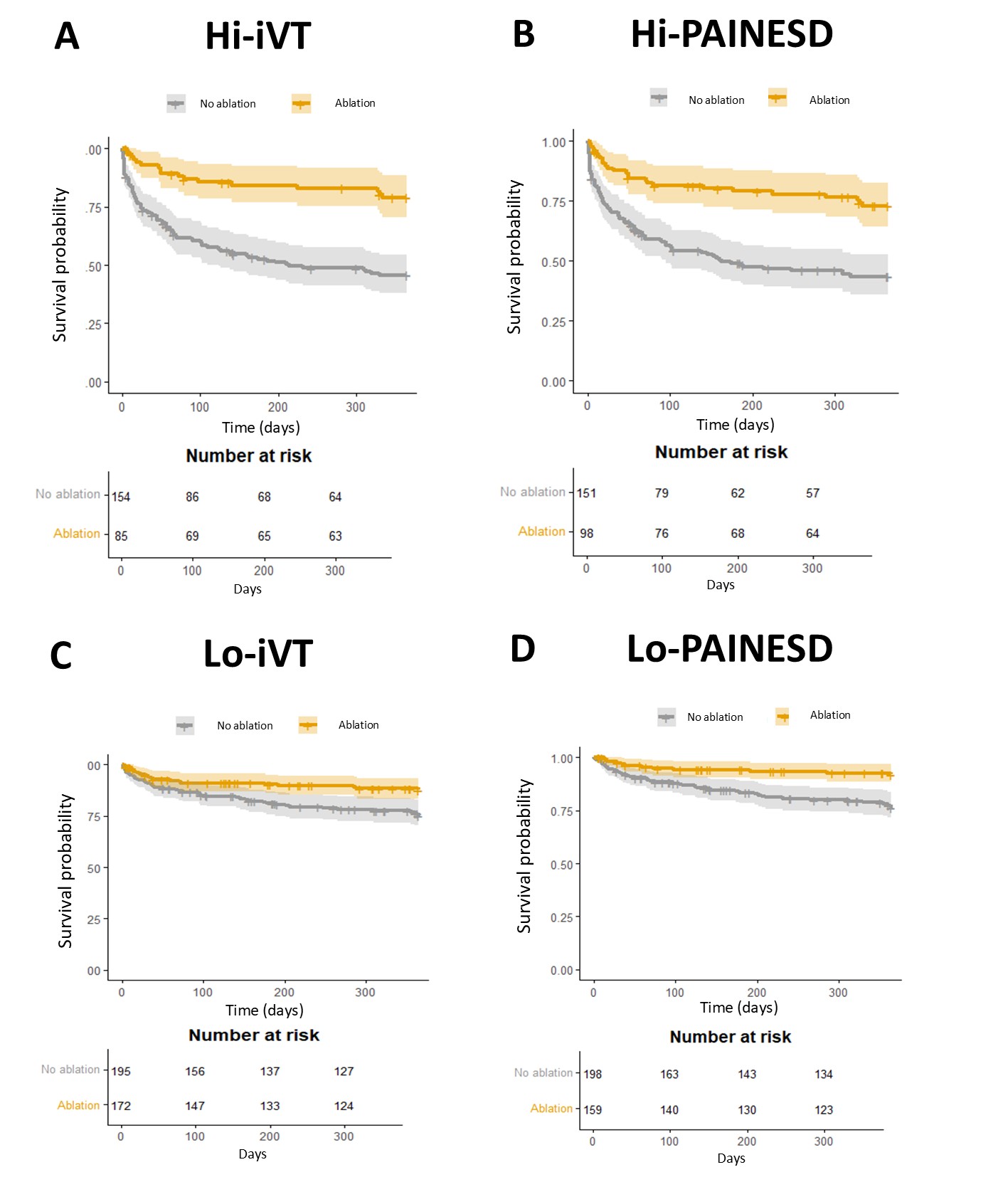
**
